# A CpG oligodeoxynucleotide enhances the immune response to rabies vaccination in mice

**DOI:** 10.1186/s12985-018-1089-1

**Published:** 2018-11-13

**Authors:** Pengcheng Yu, Jianghong Yan, Weicheng Wu, Xiaoyan Tao, Xuexin Lu, Shuqing Liu, Wuyang Zhu

**Affiliations:** Key Laboratory for Medical Virology, Ministry of Health, National Institute for Viral Disease Control and Prevention, Chinese Center for Disease Control and Prevention, Beijing, China

**Keywords:** CpG ODN, HDCV, Immunization

## Abstract

**Background:**

Rabies is a fatal disease that is preventable when post exposure prophylaxis (PEP) is administered in a timely fashion. CpG oligodeoxynucleotides (ODNs) can trigger cells that express Toll-like receptor 9, and their immunopotentiation activity in an inactivated aluminum-adjuvanted rabies vaccine for dogs has been identified using mouse and dog models.

**Methods:**

A human diploid cell rabies vaccine (HDCV) of humans and a CpG ODNs with cross-immunostimulatory activity in humans and mice were used to evaluate the immunogenicity and protective efficacy of CpG ODN in a mouse model that simulates human PEP.

**Results:**

HDCV combined with CpG ODN (HDCV–CpG) stimulated mice to produce rabies virus-specific neutralizing antibody (RVNA) earlier and increased the seroconversion rate. Compared with HDCV alone, either HDCV–1.25 μg CpG or HDCV–5 μg CpG increased the levels of RVNA. In particular, 5 μg CpG ODN per mouse significantly boosted the levels of RVNA compared with HDCV alone. IFN-γ producing splenocytes generated in the HDCV-5 μg CpG group were significantly increased compared to the group treated with HDCV alone. When the immunization regimen was reduced to three injections or the dose was reduced to half of the recommended HDCV combined with CpG ODN, the RVNA titers were still higher than those induced by HDCV alone. After viral challenge, 50% of mice immunized with a half-dose HDCV–CpG survived, while the survival rate of mice immunized with HDCV alone was 30%.

**Conclusions:**

The immunopotentiation activity of CpG ODNs for a commercially available human rabies vaccine was first evaluated in a mouse model on the basis of the Essen regimen. Our results suggest that the CpG ODN used in this study is a potential adjuvant to rabies vaccines for human use.

**Electronic supplementary material:**

The online version of this article (10.1186/s12985-018-1089-1) contains supplementary material, which is available to authorized users.

## Introduction

Rabies is caused by lyssaviruses and is almost always fatal following the onset of clinical symptoms. Approximately 59,000 humans die from rabies each year [1]. Pre- or post-exposure rabies vaccination is the only treatment to prevent rabies in humans. Rabies is 100% preventable by timely administration of post exposure prophylaxis (PEP) to bite victims, but fatalities still occur in many countries in which rabies is endemic [2–4]. In China, more than 12 million people every year are inoculated with the rabies vaccine for pre-exposure prophylaxis (PrEP) or PEP [5]. The development of more effective and productive rabies vaccines for human use is urgently needed because of the high incidence of rabies and the large dosages needed for current pre- or post-exposure rabies vaccination.

Prior to 1980, the rabies vaccine for human use was produced from sheep brain tissue and induced severe adverse effects such as neuroparalysis and encephalomyelitis. In 1980, China abandoned the animal brain tissue vaccine in favor of a cell-culture vaccine. Presently, the widely used rabies vaccines for human prophylaxis in China are cell-culture vaccines, such as the human diploid cell rabies vaccine (HDCV), purified chicken embryo cell rabies vaccine (PCECV), purified Vero cell rabies vaccine (PVRV) [6] and the primary hamster kidney cell culture vaccine (PHKCV). In our study, we used HDCV which contains the Pitman-Moore strain of rabies virus grown on MRC-5 human diploid cell culture and is concentrated by ultrafiltration and inactivated with ß-propiolactone.

An adjuvant is added to a vaccine to boost the immune response to induce higher levels of antibodies and longer-lasting protection, which can consequently reduce the amount of vaccine required. Aluminum is the first adjuvant approved by the U.S. Food and Drug Administration (FDA) for human vaccines, which was once used for improving the immunogenicity of human rabies vaccines such as PHKCV. An effective rabies vaccine should induce sufficient early RVNA to arrest rabies virus infection. Although the aluminum adjuvant can improve antibody titers to the rabies vaccine, it is argued to delay the early production of antibodies, which may be very unfavorable for the prevention of rabies [7]. As a result, and the China Food and Drug Administration banned the use of aluminum hydroxide (Al(OH)_3_) adjuvant for the human rabies vaccine on June 30, 2005. Therefore, identifying a safe, effective, and selective adjuvant for rabies vaccines is necessary.

CpG oligodeoxynucleotides (CpG ODNs) are short synthetic single-stranded DNA molecules containing unmethylated CpG dinucleotides in particular sequence contexts (CpG motifs). CpG ODNs can trigger cells that express Toll-like receptor 9 (including human plasmacytoid dendritic cells and B cells) to mount an innate immune response characterized by the production of T helper 1 and proinflammatory cytokines [8]. CpG ODNs coadministered with vaccines improve the function of professional antigen-presenting cells and boost the generation of humoral and cellular vaccine-specific immune responses [9–14]. It was reported that CpG ODNs could facilitate more vigorous RVNA responses to an inactivated aluminum-adjuvanted rabies vaccine in mice and dogs [15, 16]. In our study, we evaluated the immunopotentiation and protective efficacy provided in mice by injecting HDCV combined with CpG ODN.

## Materials and methods

### CpG ODN

The ODN (5′–TCG ACG TTC GTC GTT CGT CGT TC–3′) used in this study was synthesized by Takara Biotech Company (Dalian, China) and was diluted with endotoxin-free water and vortexed until completely dissolved.

### Cells and animals

BSR cells (a cloned baby hamster kidney cell line) were cultured at 37 °C in a 5% CO_2_ humidified incubator and maintained in Dulbecco’s minimum essential medium (Gibco, Waltham, MA USA) supplemented with 10% (*v*/v) heat inactivated fetal bovine serum (FBS, Gibco) and 1% (v/v) antibiotics (100 IU of penicillin/ml and 100 IU of streptomycin/ml, Gibco).

Specific pathogen-free (SPF) female BALB/c mice were purchased from the Vital River Laboratory Animal Technology Co., Ltd., Beijing and kept under individual ventilated cages (IVC) for all experiments. All animals were treated according to the regulations of Chinese law and the Animal Experimental Ethical Inspection of the National Institute for Viral Control and Prevention of China CDC.

### Vaccines, standards, and viruses

Freeze-dried HDCV for human use was produced by Chengdu Kanghua Biological Products Co., Ltd. For use, HDCV containing at least 2.5 IU rabies virus was reconstituted with 1.0 ml of water for injection.

A national reference standard serum (30 IU/ml) was purchased from the National Institute for Biological Standards and Control (UK). Anti-rabies nucleoprotein antibody labeled with fluorescein isothiocyanate was purchased from Fujirebio Diagnostics, Inc. The standard challenge virus (CVS-11) was provided by the National Institutes for Food and Drug Control, China. CVS-11 was propagated in BSR cells and the median lethal dose (LD_50_) in BALB/c mice was previously determined by the intramuscular (i.m.) injection route.

### MTT colorimetric assay for the activity of ODNs

A 3-(4,5-dimethylthiazol-2-yl)-2,5-diphenyltetrazolium bromide (MTT) cell proliferation and cytotoxicity assay kit (Solarbio, Beijing, China) was used to detect the activity of CpG ODN. Splenocytes from female BALB/c mice were stimulated with 6 μl of CpG ODN (100 μg/ml) or PBS and incubated for 36 h at 37 °C in 5% CO_2_. One hundred microliters of supernatant was replaced by fresh medium, and 10 μl MTT (5 mg/ml) was then added and incubated for 4 h. The supernatant was discarded, 150 μl dimethylsulfoxide was added, and cultures were shaken gently to dissolve the crystals. The absorbance of each well at 570 nm was measured using a plate reader.

### Immunization

Mice (6–8 weeks old, weight 18–22 g) were immunized by i.m. injection to the right leg of 100 μl of HDCV diluted 50 times [17]. The preparations were injected into mice using the Essen regimen (five separate doses on days 0, 3, 7, 14, and 28), which is one of the PEP schedules recommended by the World Health Organization (WHO) [18]. Serum samples were collected and separated by centrifugation at 4000 rpm for 10 min and stored at − 20 °C until testing. The RVNA titers were evaluated using the rapid fluorescent focus inhibition test (RFFIT), a standardized test recommended by the WHO.

### Evaluation of cellular immune response

Mice were sacrificed at day 14 after the first immunization, and splenocytes were prepared. The frequency of IFN-γ-secreting cells was analyzed using commercial mouse IFN-γ ELISPOT kits (BD, America) following the manufacturer’s instructions. Spots were counted using an automated ELISPOT reader (BioSystems). The mean spot number ± SD of triplicate wells for each stimulation antigen or control was calculated.

### Rapid fluorescent focus inhibition test

Challenge virus standard (CVS-11), at the dose that caused 80% infection of BSR cells after 24 h, was incubated with serial dilutions of the sera to be titrated. A reference serum (30 IU/ml) was included in each test. After 1 h of incubation at 37 °C, BSR cells were added to each well. After 24 h of incubation at 37 °C in 5% CO_2_, the percentage of infected cells at each serum dilution was estimated. This allowed the determination of the titer of the unknown neutralizing antibodies by comparison with the reference serum. The titer of the neutralizing antibodies in the sera was recorded in IU/ml, which is the global standard. Serum with a neutralizing antibody titer ≥0.5 IU/ml was considered positive.

### Statistical analysis

Statistical analysis and graphing were performed using commercially available software (GraphPad Prism 5.0). Specifically, unpaired two-tailed Student’s t-tests were applied to analyze the data and to evaluate the antibody levels expressed as geometric mean titers ± standard deviation (GMT ± S.D.). A *P*-value < 0.05 was considered statistically significant.

## Results

### Activity of CpG ODN for mouse splenic lymphocytes

An MTT cell proliferation and cytotoxicity assay kit was used to assess the activity of CpG ODN. The kit defines the stimulation index (SI) as the average value of the optical density (OD) in experimental wells divided by the average OD value in control wells. SI > 1.5 was considered to indicate specific proliferation of lymphocytes [19]. The average ODs of the CpG ODN group and the PBS group were 1.581 and 0.827, respectively. Therefore, the SI was 1.91, indicating that CpG ODN could stimulate the splenocytes in vitro, thereby enhancing the immune response.

### Immunogenicity of different doses of HDCV–CpG

To determine the enhancing effect of CpG ODN on the immunogenicity of the HDCV rabies vaccine, female BALB/c mice (*n* = 10) were immunized i.m. with HDCV combined with different doses of CpG ODN (1.25 μg, 5 μg, or 20 μg per mouse) using the Essen regimen. Serum samples were collected on days 0, 6, 8, 10, 14, 45 and 90. The RVNA in the sera of immunized mice was detected using the RFFIT method and reported as IU/ml. The preimmune sera of the mice were used as negative controls.

**Fig. 1:**
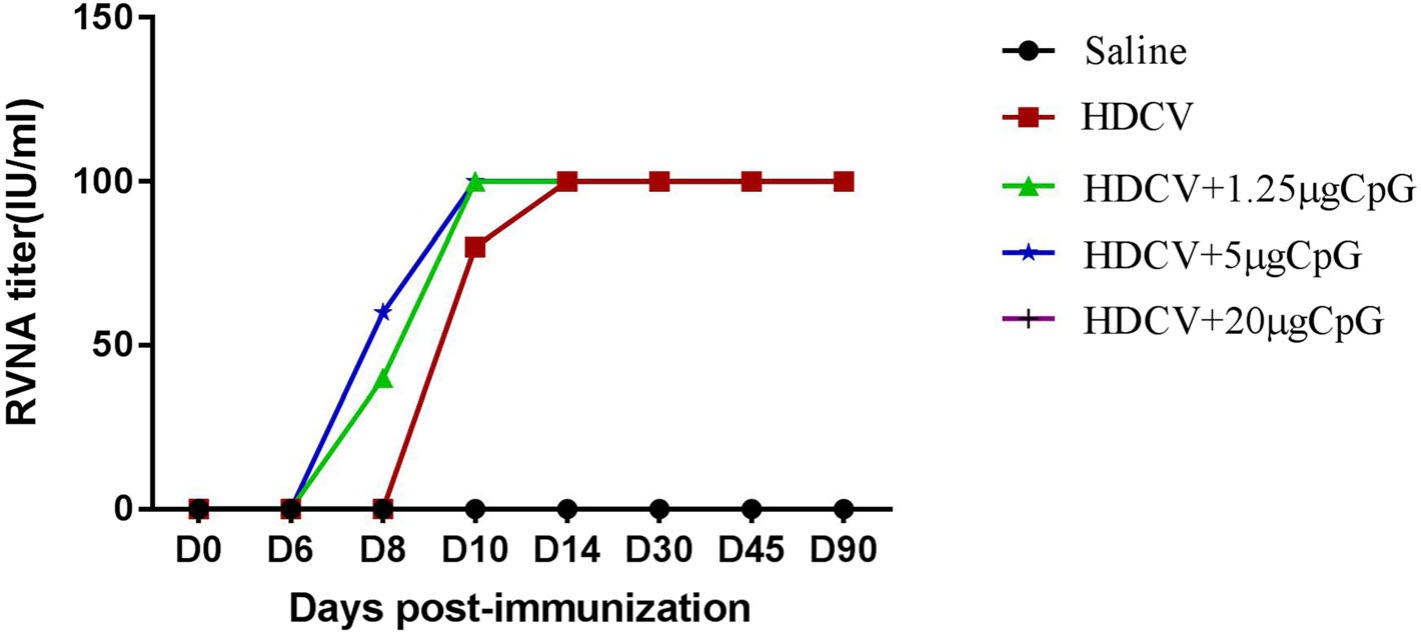
Positive conversion by different doses of CpG combined with RV. HDCV, Saline, and HDCV plus CpG at 1.25, 5 and 20 μg, respectively, were used as vaccines. Balb/c mice (*n* = 10) were immunized i.m. with the above vaccines on D0, 3, 7, 14, and 28. Serum samples were collected on D0, 6, 8, 10, 14, 30, 45 and 90. RFFIT was used to detect RVNA in the sera of mice

Seroconversion means the RVNA titer is equal to or greater than 0.5 IU/ml, which is regarded as an adequate immune response for protection by the WHO. As shown in Fig. 1, on D8 and D10 after the first immunization, the rates of seroconversion in the HDCV+ 1.25 μg CpG and HDCV+ 5 μg CpG groups were 40 and 60%, respectively, while those in the HDCV alone and HDCV+ 20 μg CpG groups were both zero. On D10, the seroconversion rates of the HDCV+ 1.25 μg CpG and HDCV+ 5 g CpG groups were both 100%, while those of the HDCV alone and HDCV+ 20 μg CpG groups were both 80%. On D14 and D45, all four groups showed 100% seroconversion. These results indicate that the CpG ODN adjuvant can stimulate mice to produce RVNA earlier and increase the seroconversion rate.

**Fig. 2:**
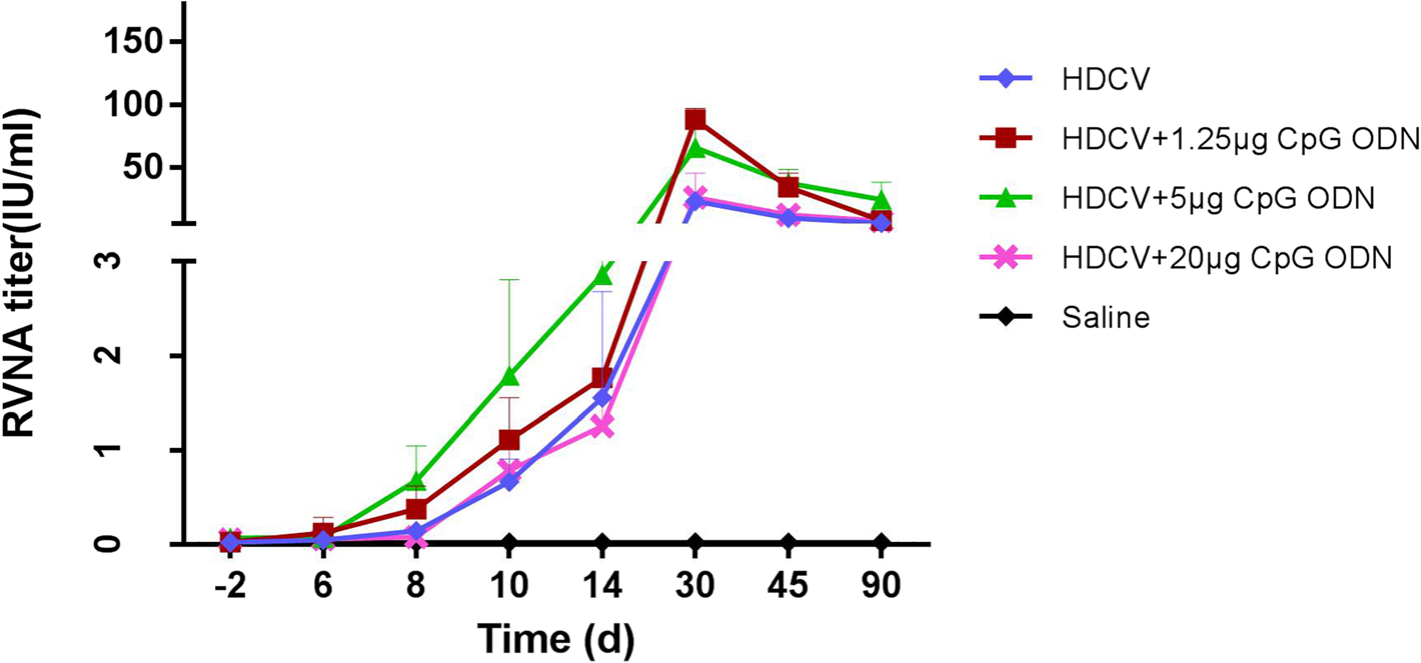
Effect of immunization on CpG combined with HDCV. HDCV, Saline, and HDCV plus CpG at 1.25, 5 and 20 μg, respectively, were used as vaccines. Balb/c mice (*n* = 10) were immunized i.m. with the above vaccines on D0, 3, 7, 14, and 28. Serum samples were collected on D0, 6, 8, 10, 14, 30, 45 and 90. RFFIT was used to detect RVNA in the sera of mice. The antibody titer was expressed in international units per milliliter (IU/ml), calculated in comparison with the national reference standard serum

As shown in Fig. 2, either HDCV–1.25 μg CpG or HDCV–5 μg CpG can increase the level of RVNA than that induced by HDCV alone. On D8 after the first immunization, the GMT of RVNA induced by HDCV+ 5 μg CpG reached 0.6 IU/ml, which was significantly different (*P* < 0.0 5) than the HDCV alone group. Consistently, HDCV+ 5 μg CpG stimulated a significant level of RVNA on D10, 14, 30, 45 and 90. Compared with the HDCV groups, the GMT of the HDCV+ 1.25 μg CpG groups also showed significant differences on D30 and D60 (*P* < 0.0 1). Compared with these two groups, the GMTs from the HDCV+ 20 μg CpG group showed no significant differences at any of the 8 points of serum collection. These results indicate that 5 μg CpG per mouse is optimal to facilitate production of a stronger RVNA response in mice.

### Cellular immune response in vaccinated mice

**Fig. 3:**
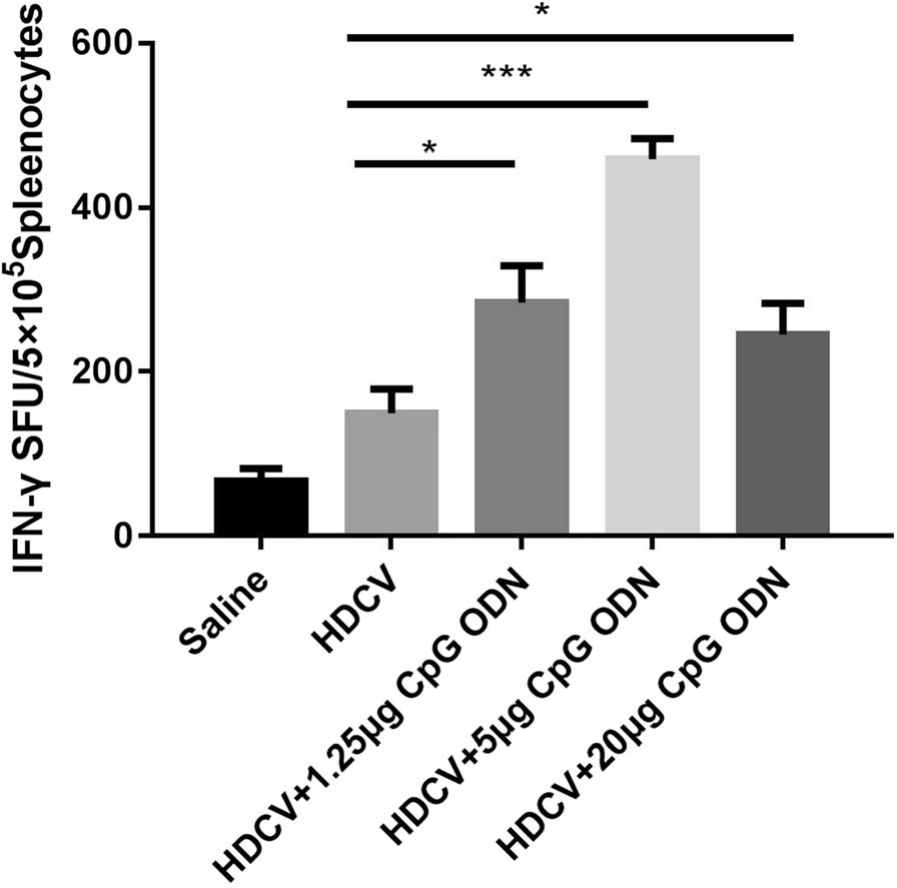
Cellular immune response in vaccinated mice. Splenocytes from immunized mice were isolated 14 days after the first immunization. The figure showes the number of IFN-γ-secreting cells as determined by ELISPOT assay. The data are expressed as the means ± SEM (*n* = 5; *P < 0.05, **P < 0.01, ***P < 0.001)

To determine whether T cells were activated, the frequencies of IFN-γ-producing cells at the single-cell level were determined by ELISPOT assay following stimulation of mouse spleen lymphocytes with HDCV and HDCV combined with CpG ODN. The HDCV-5 μg CpG group generated approximately 500 SFU/5 × 10^5^ of IFN-γ-specific splenocytes (Fig. 3**)**, which was significantly higher than the group treated with HDCV alone. Similarly, the numbers of IFN-γ-secreting cells generated in mice primed with HDCV+ 1.25 μg CpG and HDCV+ 1.25 μg CpG groups were also significantly different from those in the HDCV group.

### RVNA response induced by reduced injections of HDCV–CpG

To evaluate the possibility of reducing the number of injections required for immunization with HDCV–CpG, mice (*n* = 10) were immunized i.m. 5 times with HDCV or 5, 4, 3, or 2 times with HDCV–CpG, reported as HDCV-5, HDCV–CpG-5, HDCV–CpG-4, HDCV–CpG-3, and HDCV–CpG-2, respectively. The HDCV-5 and HDCV–CpG-5 groups were immunized using the Essen regimen. The HDCV–CpG-4 group was injected on D0, 3, 7 and 14; the HDCV–CpG-3 group, on D0, 3 and 7; and the HDCV–CpG-2 group on D0 and 3. The dose of CpG ODN used was 5 μg/mouse. On D35 after the first immunization, sera were collected from the retroorbital sinus of mice, and RVNA in the sera was measured using RFFIT.

**Fig. 4:**
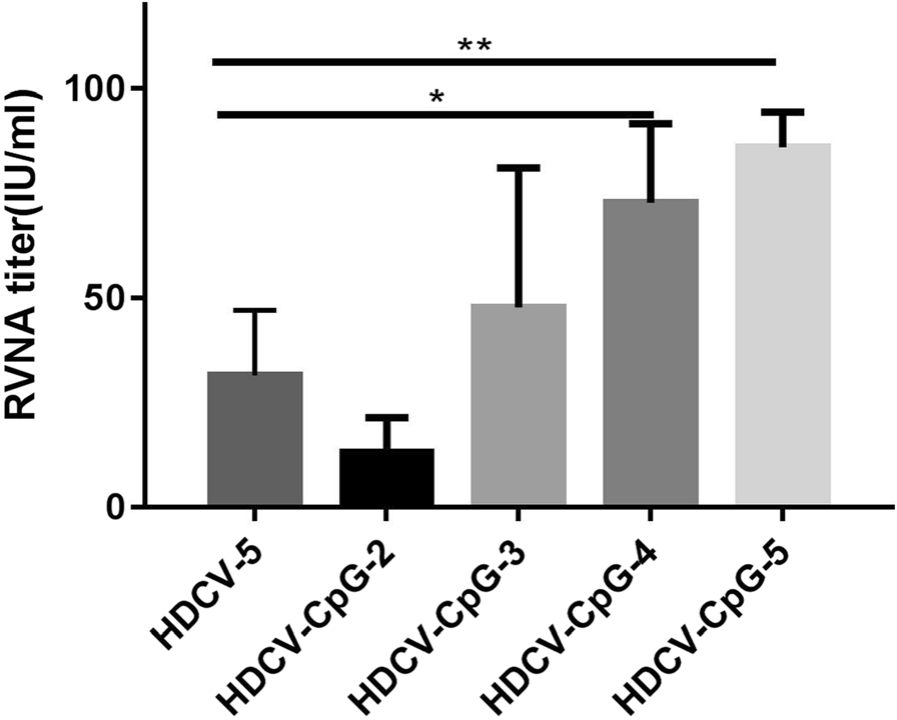
RVNA response induced by reduced injections of HDCV-CpG. Balb/c mice (*n* = 10) were immunized i.m. with HDCV for 5 injections and HDCV plus CpG for 5, 4, 3 and 2 injections. Serum samples were collected on D35. RFFIT was used to detect RVNA in the sera of mice. The antibody titer was expressed in international units per milliliter (IU/ml), calculated in comparison with the national reference standard serum

As shown in Fig. 4, the GMTs of the HDCV–CpG-5 and HDCV–CpG-4 groups were all significantly higher (*P* < 0.05) than that of HDCV-5 group, which was injected five times with HDCV alone. HDCV–CpG-3 stimulated mice to produce similar levels of RVNA similar to those of the HDCV-5 group, and there was no statisticant difference between the GMTs from the two groups (*P* > 0.05). Although the RVNA titer of the HDCV–CpG-2 group was lower than that of HDCV-5, all tested sera were above 0.5 IU/ml. These results indicate that CpG ODN can enhance RVNA production in response to HDCV and maintain similar RVNA levels even when the number of injections is reduced to two.

### RVNA response induced by a reduced dose of HDCV–CpG

To evaluate the possibility of reducing the immunizing dose of HDCV–CpG, mice (*n* = 10) were immunized i.m. with HDCV alone or with CpG ODN combined with a full dose of HDCV, a half dose of HDCV, a quarter dose of HDCV or an eighth dose of HDCV, reported as HDCV, HDCV–CpG, 1/2HDCV–CpG, 1/4HDCV–CpG, and 1/8HDCV–CpG, respectively. All groups were immunized using the Essen regimen, and the dose of CpG ODN was 5 μg/mouse. On D35 after the first immunization, sera were collected and the RVNA titers in the sera were measured by RFFIT.

**Fig. 5:**
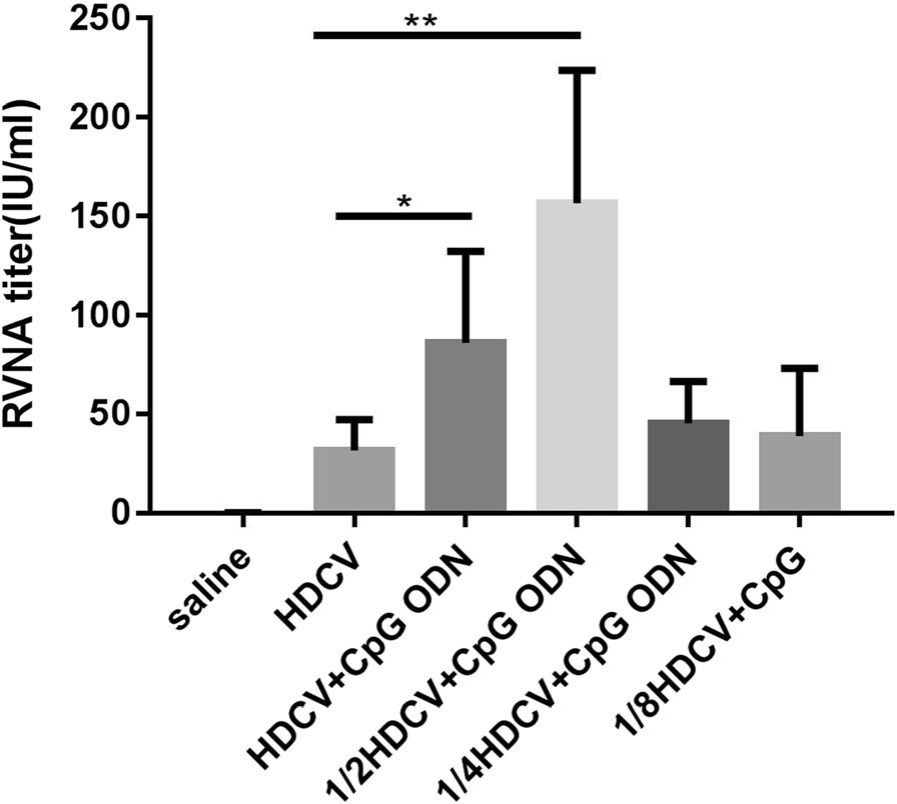
RVNA response induced by reduced doses of HDCV-CpG. Balb/c mice (*n* = 10) were immunized i.m. with Saline, HDCV, or HDCV (1, 1/2, 1/4 and 1/8 dose) plus CpG for 5 injections. Serum samples were collected on D35. RFFIT was used to detect RVNA in the sera of mice. The antibody titer was expressed in international unit per milliliter (IU/ml) calculated in comparison with the national reference standard serum

As shown in Fig. 5**,** a half dose of HDCV with CpG induced the highest RVNA titers of all tested groups, which was significantly higher than that in the group immunized with HDCV alone (*P* < 0.05). Even when the dose of HDCV was reduced to one-eighth, the RVNA titer was positive in all mice and not significantly different from that in the HDCV group (*P* > 0.05). This result indicate that CpG ODN can enhance the immunogenicity of HDCV and stimulate mice to produce similar RVNA levels even if the dose of HDCV is reduced to one-eighth.

### Protection of immunized animals against rabies virus challenge

To test whether the HDCV–CpG could elicit adequate protective immunity against a robust rabies virus challenge in an animal model, CVS-11 was prepared for infection at 50 LD_50_ on the hind leg. After 2 h, all groups (Saline, HDCV, HDCV+CpG, HDCV+ 1/2 CpG, HDCV+ 1/4 CpG and HDCV+ 1/8 CpG) were immunized with 5 injections. BALB/c mice in all test groups behaved normally for 6 days after the infection. Infected mice did not survive once clinical symptoms such as decreased activity, clumsiness, poor coordination, decreased food intake and anxiety, appeared between day 7 and 16, and all mice died within 4–6 days after the onset of abnormal symptoms. Densely distributed florescent spots were observed in the direct fluorescence antibody test, indicating that death was caused by rabies viral infection of the central nervous system.

**Fig. 6:**
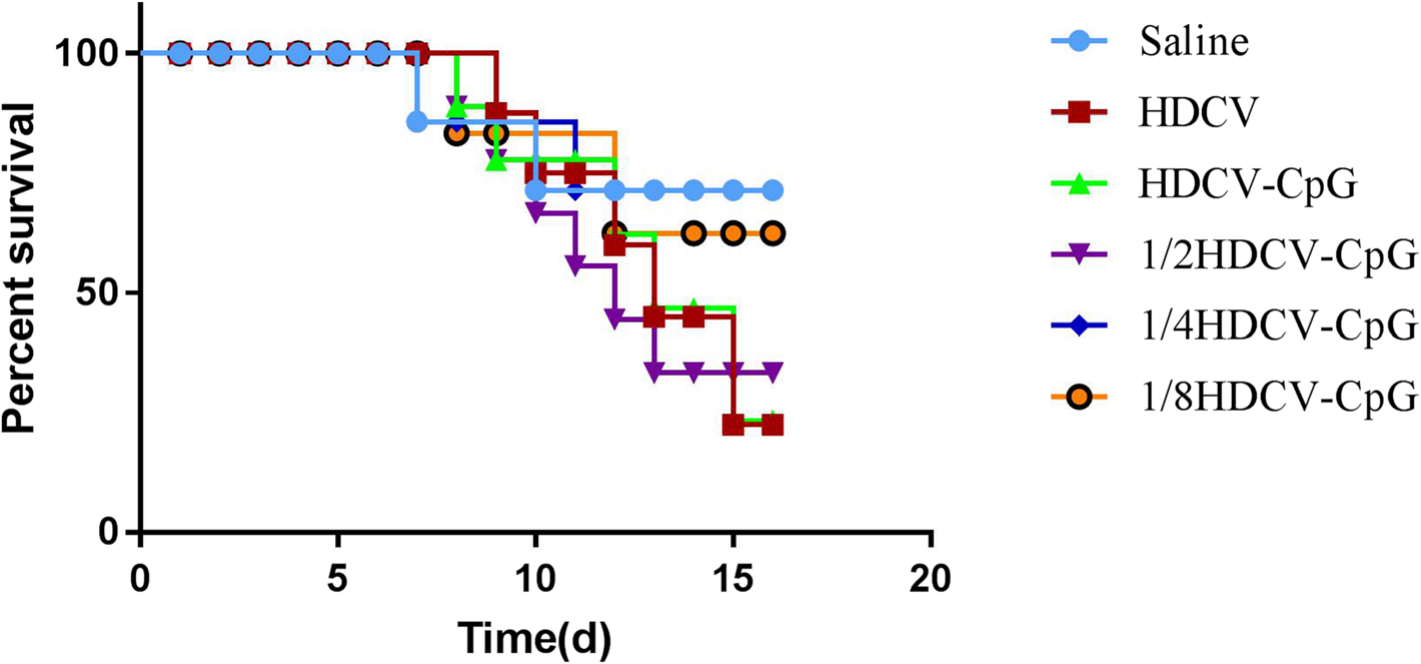
Survival curve of different doses of HDCV combined with CpG. Balb/c mice (n = 10) were injected i.m. with CVS-11. On D0 of exposure, mice were immunized with HDCV or HDCV (1, 1/2, 1/4 and 1/8 dose) plus CpG for 5 injections

The survival rates for all test groups are shown in Fig. 6. The majority (70%) of mice in the HDCV group died after viral challenge, whereas the HDCV–CpG group was better protected, with only 50% of mice dying. Furthermore, the survival rates of the 1/2HDCV–CpG group (40%) and the 1/4HDCV–CpG group (40%) were both higher than that of the HDCV group (30%). These results indicated that HDCV combined with CpG ODN stimulates mice to produce RVNA earlier, which protects the mice from early death.

## Discussion

CpG ODNs have been reported to boost the humoral immune response induced by vaccines against a large number of pathogens, including anthrax, *Leishmania*, influenza virus, measles virus, lymphocytic choriomeningitis virus, orthopoxviruses, hepatitis B surface antigen, and tetanus toxoid, improving antigen-specific antibody titers by up to three orders of magnitude [20–28]. Several studies have indicated that CpG ODNs accelerate the development of vaccine-induced responses. For example, mice vaccinated with CpG-adjuvanted anthrax vaccine-adsorbed (AVA) developed protective immunity three times faster than those immunized with AVA alone, with significant protection observed within 5 versus 15 days (*P* < 0.05). The combination of CpG ODN with AVA accelerated the serum IgG anti-*Bacillus anthracis* (BA) response, yielding serum anti-BA titers that were ten-fold higher and significantly more protective by day 10 (*P* < 0.05) [29]. In another study, administering CpG ODN with recombinant herpes simplex virus (HSV)-1 glycoprotein B intranasally induced significant levels of glycoprotein B-specific IgA and anti-HSV cytotoxic T lymphocytes in the genital tract and protected mice from genital HSV challenge [30].

Studies have shown that the rabies virus attacks the nervous tissue and appears to replicate almost exclusively in neuronal cells. Once introduced through the skin or mucous membrane, the virus begins to replicate in striated muscles at the wound site. To block the rapid replication and migration of rabies virus, vaccines should be administered to activate the immune system as soon as possible. Especially when the bite is on the face or head, the early induction of RVNA is important to save lives. Unlike other diseases, rabies is almost always fatal but preventable with an incubation period of 1–3 months. This feature allows time for post-exposure treatment with the rabies vaccine to induce RVNA and to neutralize the rabies virus. Therefore, it is important to induce an earlier and higher RVNA response with rabies vaccines. In this study, we demonstrated that HDCV combined with CpG ODN could improve the seroconversion rate and RVNA titer in a mouse model. In particular, 5 μg CpG per mouse was optimal to enhance immunopotentiation of HDCV because HDCV–5 μg CpG could increase the seroconversion rate and produce significantly higher RVNA levels in mice. Interestingly, the HDCV+ 20 μg CpG group had a lower rate of seroconversion and lower GMT of RVNA, perhaps due to damage caused by the high dose of CpG to the spleen (Additional file 1: Figure S1), which is the most important organ for humoral immunity. In addition, we observed that even one-quarter of the recommended dose of HDCV combined with CpG ODN induced better protection than HDCV alone. This result also indicated that in mice exposed to rabies virus that were administered HDCV combined with CpG ODN, RVNA was produced earlier and at higher levels than following immunization with HDCV alone.

Previous studies have shown that the TLR-9 molecule expressed by humans and mice differs by 24% at the amino acid level, and the cells that express TLR-9 vary between these species [31–34]. The motif (consisting of a CpG dinucleotide plus flanking regions) that optimally stimulates immune cells differs between mice and humans [35–38]. Although differences exist, another study demonstrated that mice may be used as an animal model to evaluate the activities of some human CpG ODN containing the 5’-GTCGTT-3′ motif [39]. The core of the sequence (−-TCGT--) of CpG ODN used in our study is the human CpG motif, which has strong activity to human immune cells. The CpG ODN with this motif had good adjuvant activity in phase I/II clinical studies of a synergistic hepatitis B prophylactic vaccine [40]. Therefore, HDCV combined with CpG ODN in a mouse model simulating human PEP can facilitate production of an earlier, stronger, and longer-lasting RVNA response and can protect mice from rabies after exposure. These results could have a potential impact on human PEP, although the animal model is not suitable for the evaluation of human vaccines. The immunopotentiation and safety of the CpG ODN used in this study to a rabies vaccine for human use should be further evaluated with clinical trials in the future.

## Conclusions

In this study, HDCV combined with CpG ODN in a mouse model simulating human PEP could facilitate production of an earlier, stronger, and longer-lasting RVNA response and protect mice from rabies after exposure to CVS-11. Further studies are needed to investigate the toxicity and side effects of CpG ODNs used in the mouse model. However, the results of this study suggest that CpG is a potential adjuvant for rabies vaccines for human use.

## Additional file

Additional file 1: **Figure S1.** Histopathological changes of mice spleen. A: HDCV; B: HDCV+ 1.25 μg CpG; C: HDCV+ 5 μg CpG group; D: HDCV+ 20 μg CpG (HE× 400). To evaluate the safety of different doses of CpG ODN, we examined the morphological changes and pathological changes using hematoxylin and eosin stained spleen tissues collected at D14. (DOCX 456 kb)

## Abbreviations

CVS: Challenge virus standard
GMT ± S.D.: geometric mean titer ± standard deviation
HDCV: human diploid cell rabies vaccine
ODN: oligodeoxynucleotid
PCECV: purified chicken embryo cell rabies vaccine
PEP: post exposure prophylaxis
PHKCV: primary hamster kidney cell culture vaccine
PVRV: purified Vero cell rabies vaccine
RVNA: rabies virus-specific neutralizing antibody
