# Supplementary material for: A CpG oligodeoxynucleotide enhances the immune response to rabies vaccination in mice
Source: Virol J. 2018 Nov 13;15:174. doi: 10.1186/s12985-018-1089-1 (PMC6234694; doi:10.1186/s12985-018-1089-1)
Supplement: Supplementary file 1 — Figure S1. Histopathological changes of mice spleen. A: HDCV; B: HDCV+ 1.25 μg CpG; C: HDCV+ 5 μg CpG group; D: HDCV+ 20 μg CpG (HE× 400). To evaluate the safety of different doses of CpG ODN, we examined the morphological changes and pathological changes using hematoxylin and eosin stained spleen tissues collected at D14. (DOCX 456 kb) [file 12985_2018_1089_MOESM1_ESM.docx]

The spleens from the HDCV+20 μg CpG group were larger than those of other groups, and showed peripheral arterial sheath thickening in the white pulp and an increased number of macrophages. In contrast, the spleens of the HDCV+1.25 μg CpG and HDCV+5 μg CpG groups showed no significant changes in spleen size, the white pulp, or the periarterial lymphatic sheath compared with the HDCV only group (**Fig. S1**). These results indicated that a high dose of CpG could injure the spleen and influence humoral immunity, meaning that the RVNA titer of the HDCV+20 μg CpG group was not higher than the other two HDCV+CpG groups.

**Fig. 1 Histopathological changes of mice spleen**

A: HDCV; B: HDCV+1.25μg CpG; C: HDCV+5μg CpG group; D: HDCV+20μg CpG (HE×400)

To evaluate the safety of different doses of CpG ODN, we examined the morphological changes and pathological changes using hematoxylin and eosin stained spleen tissues collected at D14.
